# Control of axillary bud growth in tobacco through toxin gene expression system

**DOI:** 10.1038/s41598-021-96976-3

**Published:** 2021-09-01

**Authors:** Jing Lv, Ya-Qiong Chen, An-Ming Ding, Bo Lei, Jing Yu, Xiao-Ming Gao, Chang-Bo Dai, Yu-He Sun

**Affiliations:** 1 Tobacco Research Institute, Chinese Academy of Agricultural Sciences, Qingdao, 266101 China; 2 Key Laboratory for Tobacco Gene Resources, State Tobacco Monopoly Administration, Qingdao, 266101 China; 3 Graduate School of Chinese Academy of Agricultural Sciences, Beijing, 100081 China; 4 Molecular Genetics Key Laboratory of China Tobacco, Guizhou Academy of Tobacco Science, Guiyang, 550081 China; 5 Kunming Tobacco Monopoly Administration, Kunming, 650000 China

**Keywords:** Expression systems, Molecular engineering, Plant biotechnology, Biological techniques, Biotechnology

## Abstract

The control of axillary bud development after removing the terminal buds (topping) of plants is a research hotspot, and the control of gene expression, like switching on and off, allows us to further study biological traits of interest, such as plant branching and fertility. In this study, a toxin gene control system for plants based on dexamethasone (DEX) induction was constructed, and the positive transgenic tobacco exhibited growth retardation in the application area (axillary bud). The expression level of the lethal Diphtheria toxin A (*DTA*) gene under different DEX concentrations at different application days was analyzed. The highest expression levels appeared at 5 days after the leaf injection of DEX. The *DTA* transcripts were induced by 5 µM DEX and peaked in response to 50 µM DEX at 5 days after leaf injection. Here, a chemical induction system, combined with a toxin gene, were used to successfully control the growth of tobacco axillary buds after topping. The *DTA* expression system under DEX induction was sensitive and efficient, therefore, can be used to control axillary bud growth and development in tobacco.

## Introduction

The growth of axillary buds determines the shoot branching and morphology of plants, and its initiation and development are regulated by a series of genetic, hormonal and environmental signals^1,2^. Two kinds of genes are involved in axillary bud formation, axillary meristem initiation-related genes, such as *GRAS*^3^, *MYB*^4^ and *NAC* transcription factor^5^, and axillary bud growth-related genes, such as *F-box* protein^6^, *KNOTTED-like* transcription factor^7^ and *SPL* transcription factor^8^.

In the field production of crop species, like cotton etc., it is necessary to decapitate or remove the terminal buds (known as topping) at the initial reproductive stage, to maximize the yield of crops. Like other crop species, axillary buds of tobacco (*Nicotiana tabacum L.*) develop and form a large number of lateral branches, owing to the disappearance of apical dominance, which affects the quality and yield of tobacco leaves after topping. Manually removing and suckercides are commonly applied on tobacco to control the axillary bud formation and growth though these methods are time-consuming, labor-intensive, expensive, and environment unfriendly.

Chemical-induced expression systems have promoters or regulatory elements that can be activated by various chemical signals (such as ethanol, tetracycline, and steroids). The expression levels of plant genes can be regulated at specific times through the purposeful use of inducing substances. Currently, three kinds of chemical-induced expression systems based on disinhibition, inactivation and activation have been constructed and applied. Among them, the glucocorticoid induction system has been widely used in tobacco, Arabidopsis, and other plants. After the application of dexamethasone (DEX) to transgenic tobacco plants containing GVG activators, the expression level of the luciferase gene was more than 100 times greater than in the control group^9^. Moreover, Arabidopsis harboring bacterial non-toxic genes (avrRpt2) induced by DEX showed non-sensitive cell death, which could provide the basis and method for studying the physiological and biochemical processes of cell death caused by non-toxic genes in susceptible and resistant plants^10^. The DEX-inducible derivative-pOp/LhGR system can be used to stringently and efficiently regulate the expression of *uidA* and the cytokinin-biosynthetic gene *ipt* in tobacco under tissue culture and greenhouse conditions^11^ without the inhibitory effects on plants that were previously reported^12–14^. At present, rare study on the regulation of plant architecture through chemical induction systems has been reported.

The use of tissue-specific promoters to control the expression levels of toxic genes can effectively regulate fertility, resistance and immunoprotective capabilities in plants^15–17^. Various types of toxin genes, including ricin, microtoxin and marine biotoxin, have not been used in the control of axillary buds. In this study, an modified toxin gene Diphtheria toxin A (*DTA*) was used for DEX induction, and the corresponding transgenic plants showed suppressed axillary bud growth and development in their architecture, which provides a basis for the further study of crop agronomic characteristics.

## Materials and methods

### Plant material, growth, and DNA extraction

The cultivated tobacco (*Nicotiana tabacum* L. cv. ‘honghuadajinyuan’) was grown at 22 °C in a standard glass greenhouse at Qingdao, Shandong Province, China. All plants(*Nicotiana tabacum* L.) are provided by Tobacco Research Institute, Chinese Academy of Agricultural Sciences(Qingdao, China) and all experiments have been carried out in accordance with relevant guidelines. The plants for testing were planted in the experimental plots under the same cultivation conditions. Total DNA from leaves was extracted using a Plant DNA Isolation Reagent (TaKaRa, Japan). Quality of DNA samples were run on 1% agarose gels, and the purity was checked using NanoDrop2000 spectrophotometer (Thermo, USA).

### qRT-PCR

Total RNA from leaves was extracted using the GeneJET Plant RNA Purification Mini Kit (MBI Fermentas, Canada). RNA samples were run on 1% agarose gels, and the quality was checked using a NanoDrop2000 spectrophotometer. The cDNA was synthesized using the RevertAid First-Strand cDNA Synthesis Kit (MBI Fermentas, Canada), and cDNA was used for fluorescence quantitative PCR analysis with a TB Green Premix Ex Taq II kit (Tli RNaseH Plus) (TaKaRa, Japan) and actin gene was used as the reference gene.

### Plasmid construction and genetic transformation

**Figure 1:**
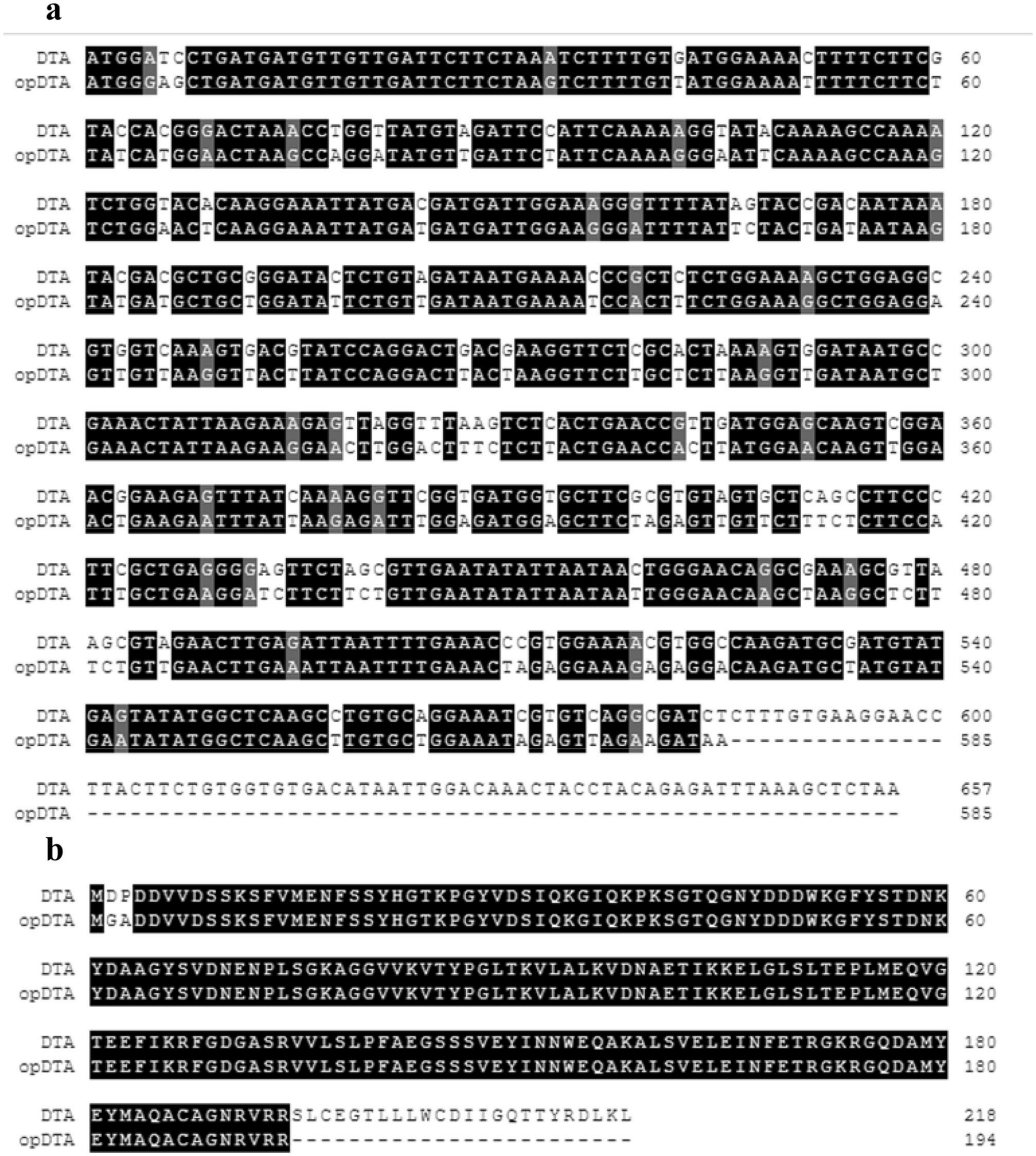
The alignment results of CDS sequences (**a**) and protein sequences(**b**) of modified *DTA* and original *DTA*.

**Figure 2:**
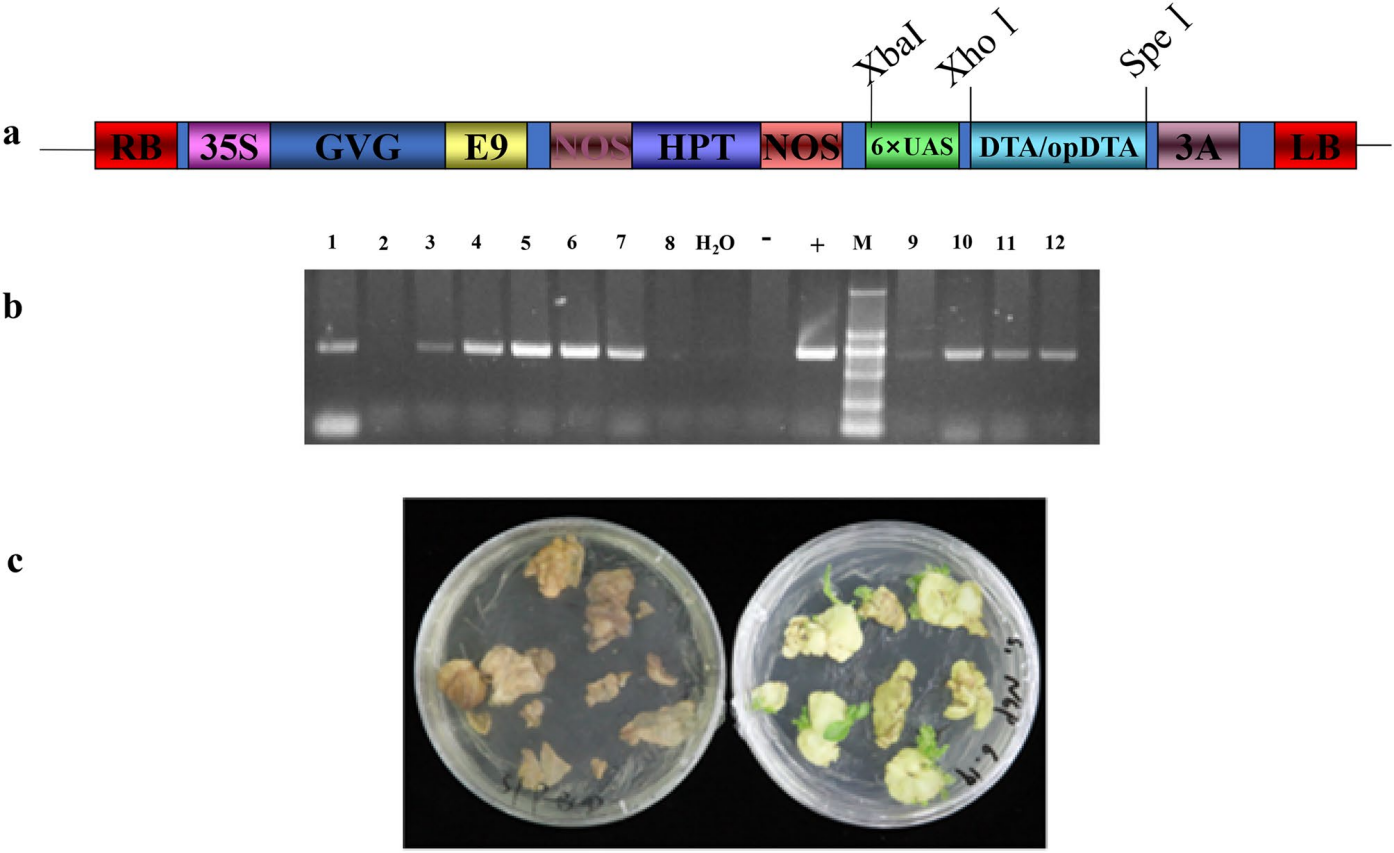
Optimized *DTA* gene exhibits weak toxicity to tobacco leaf discs in DEX-inducible system. (**a**) Schematic diagram of the recombination vector pTA7002::DTA and opDTA; (**b**) PCR assay to detect the opDTA gene in T0 transgenic plants; c: leaf disc of tobacco after infecting by Agrobacterium tumefaciens harboring pTA7002::DTA (left) and optimized pTA7002::opDTA (right).

The CDS sequence (GenBank: KY766997.1) of the toxic gene *DTA* ^18^ was optimized and synthesized by The Beijing Genomics Institute (BGI) . The alignment results of CDS sequences and protein sequences of optimized *DTA* (opDTA) and original *DTA* were shown in Fig. 1. The original and optimal DTA sequence was cloned into restriction enzymes *XhoI* and *SpeI* site of the pTA7002 vector(Fig. 2a) ^19^. The resultant constructs was then transformed into *Agrobacterium tumefaciens* strain EHA105 ^20^ and introduced into cultivated tobacco ‘honghuadajinyuan’ plants using the leaf disc method ^21^. Transgenic plants were selected on Murashige and Skoog (MS) Medium containing 100 µg/ml hygromycin. Positive transgenic plants were selected by PCR using the specific primers pTA7002-F(5’-TTGCATGCCGGTCGACTCTA-3’) and DTA-R (5’-GAGCCTTAGCTTGTTCCCAA-3’).

### DEX application and morphological observations

For DEX treatments, the solutions containing 5, 25, 50 and 100 µM DEX were injected into the same position of tobacco leaves through a pinhole. Total RNA was then separately extracted from the injection leaves after 1-, 3-, 5- and 10-days treatment. The expressional levels of opDTA at four time points were determined by qRT-PCR with the primers DTAYF (5’-TGGGAGCTGATGATGTTGTTGA-3’) and DTAYR (5’-ACTCCTCCAGCCTTTCCAGA-3’) as described above. The lanolin containing 50 µM DEX was applied to the axils of the first leaf after topping during the vigorous growth period, the treated plants were then photographed at different times. The leaf length, leaf width and leaf weight of cultivated tobacco with different treatments after topping were measured separately in 90-day-old plants, each sample was measured by 5 replicates.

## Results

### Optimization of the toxic gene and inducible system for tobacco regeneration

To investigate the effect of toxic gene on axillary bud of tobacco, the pTA7002::DTA inducible system was constructed and subsequently transformed into common tobacco through leaf disc method (Fig. 2a). Since the inducible system with original *DTA* is extremely toxic to tobacco leaf disc even without DEX treatment^22^ (Fig. 2c), we modified *DTA* gene according to codon preference of tobacco, and removed the last 24 amino acids, produced pTA7002::opDTA vector (Fig. 1). Though the explants were pale during antibiotic selection, we successfully obtained positive transgenic plants with lower regeneration efficiency (Fig. 2b), suggesting that the toxic inducible system with optimized *DTA* is suitable for tobacco transformation (Fig. 2c).

### Optimization of hormone application time and application concentration

**Figure 3:**
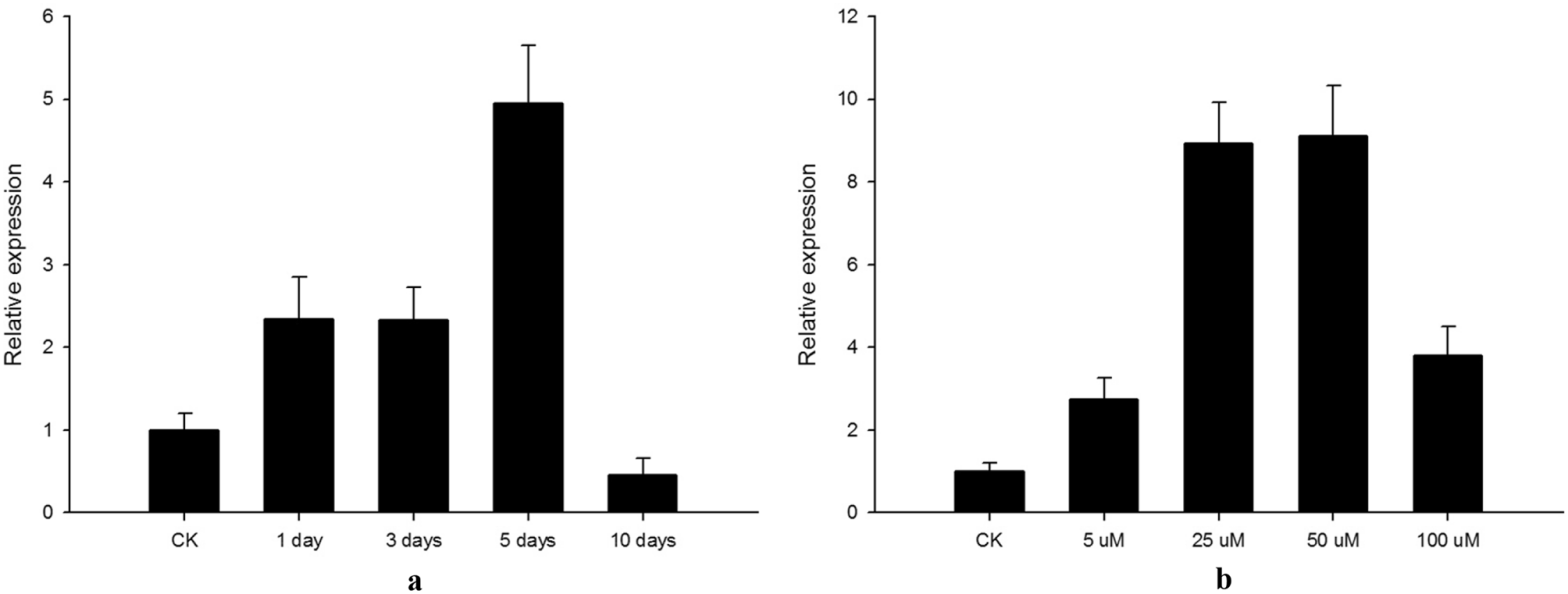
Transcriptional levels of *DTA* gene on the first axillary buds applied with different concentrations and time duration. (**a**) Changes in the expression level of the Diphtheria toxin A gene at 10 days after 50 µM dexamethasone induction; b: Changes in the expression level of the Diphtheria toxin A gene 5 days after induction with different dexamethasone concentrations.

To confirm the optimal hormone application time and concentration, the fluorescence quantitative PCR were performed to investigate the *DTA* expression level. As shown in Fig. 3a, the transcriptional level of DTA after 1 day treatment, was 1.5-fold higher than control. The highest induction level was obtained at 5 days after DEX application (Fig. 3a), and then declined. Similarly, 5 days after application, the *DTA* expression level increased as the DEX concentration increased. It peaked at 50 µM and then declined (Fig. 3b).

### The growth of axillary bud of first leaf was inhibited by toxin gene expression system

**Figure 4:**
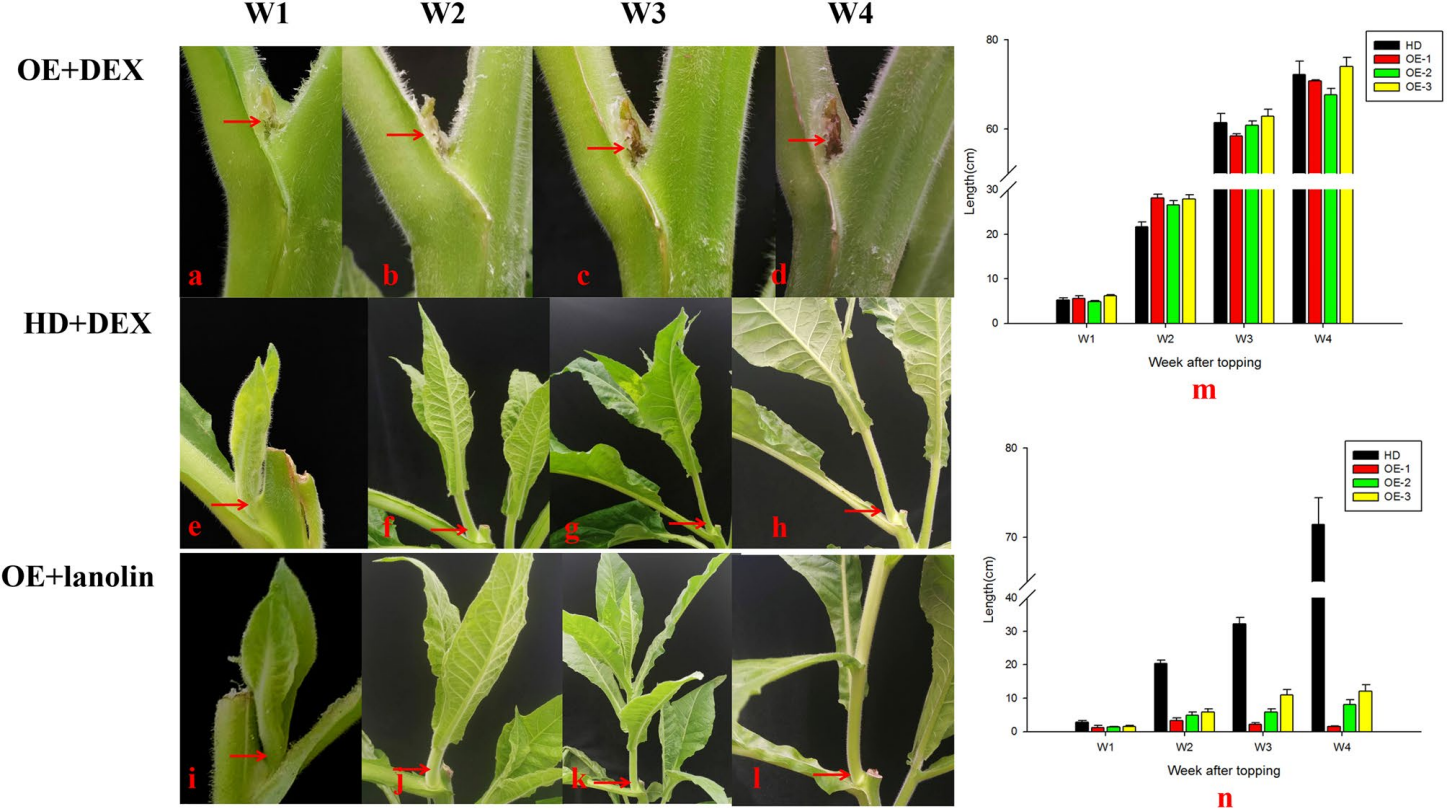
Growth of the axillary buds of the first leaf after topping in positive transgenic and control plants. (**a**–**d**): growth of the axillary buds at the application site treated with both lanolin and 50 µM dexamethasone in positive transgenic plants (from left to right: 1, 2, 3 and 4 weeks after topping); (**e**–**h**): growth of the axillary buds at the application site treated with both lanolin and 50 µM dexamethasone in control plants (from left to right: 1, 2, 3 and 4 weeks after topping); (**i**–**l**) growth of the axillary buds at the application site treated with lanolin in positive transgenic plants (from left to right: 1, 2, 3 and 4 weeks after topping); (**m**) length of the axillary buds at the application site treated with lanolin after topping in control plants and 3 positive transgenic lines; (**n**) length of the axillary buds at the application site treated with both lanolin and 50 µM dexamethasone after topping in control plants and 3 positive transgenic lines.

Based on the investigation of hormone application concentrations, lanolin containing 50 µM DEX was applied to the axillary buds of the first leaf after topping in positive transgenic seedlings. Two treatments were used as controls: lanolin without DEX was applied to the axillary buds of the first leaf after topping in positive transgenic seedlings and lanolin containing 50 µM DEX was applied to the axillary buds of the first leaf after topping in cultivated tobacco plants. The growth of axillary buds from the first leaf after topping was observed, measured, and photographed (Fig. 4a–n). As is shown in Fig. 4, when lanolin without DEX was applied to transgenic positive seedlings and lanolin containing DEX was applied only to negative controls, the axillary buds at the application sites developed normally after topping, whereas the development of axillary buds at the application sites receiving both lanolin and 50 µM DEX were blocked after topping in positive transgenic seedlings, which indicated that the toxin gene *DTA* induced by DEX plays a role in inhibiting the growth of axillary buds. The growth inhibition was maintained for 4 weeks in transgenic tobacco compared with that in normal plants (Fig. 4m–n), shown that the inducible system was suitable and stable for inhibition of axillary bud development in plants.

### The accurate control of axillary bud development by toxin gene expression system

**Figure 5:**
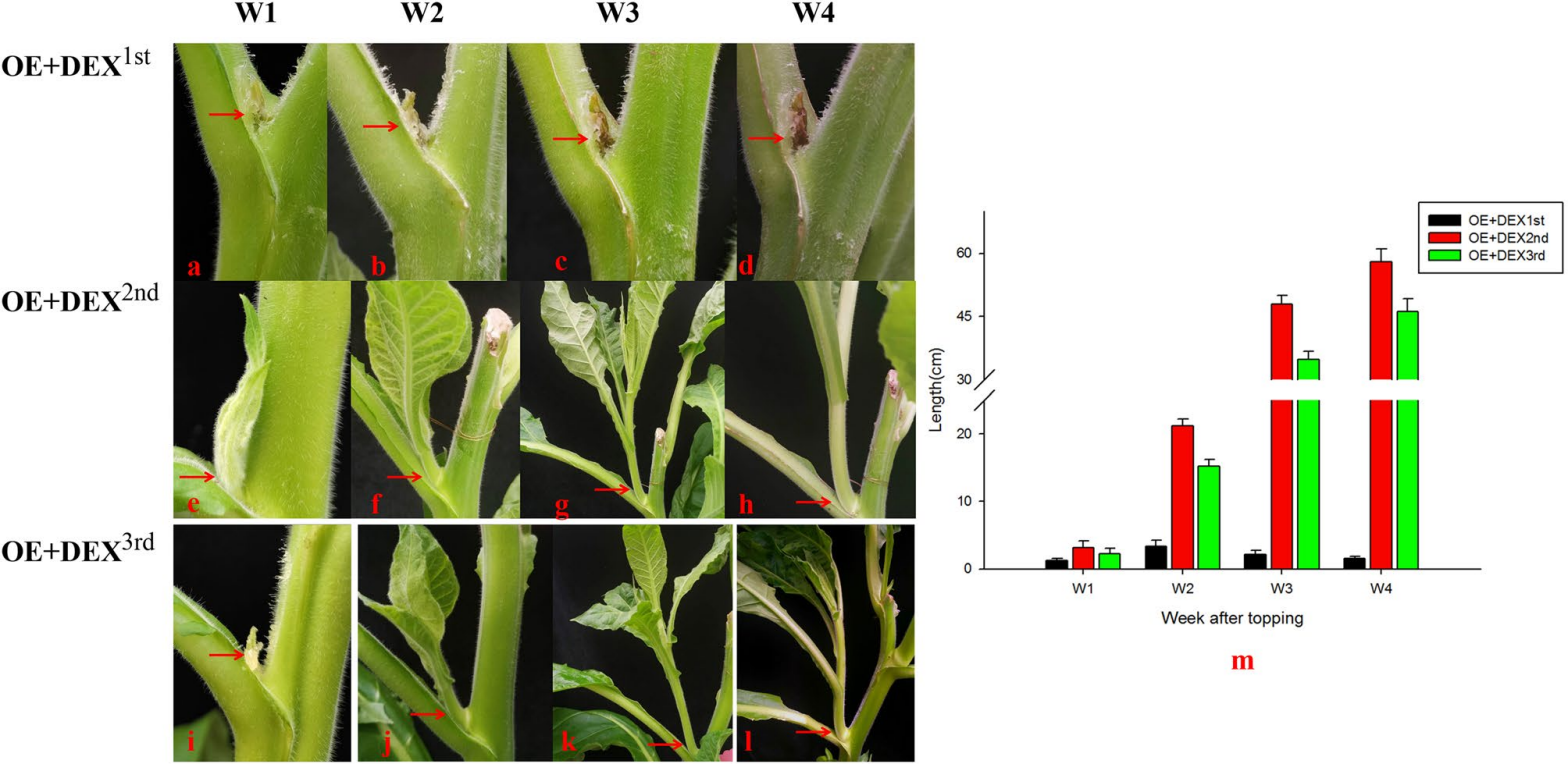
Growth of the axillary buds of the 1st, 2nd and 3rd leaves after topping of positive transgenic plants. (**a**–**d**) Growth of the axillary buds of the 1st leaf at 1, 2, 3 and 4 weeks after topping as shown in Fig. 4; (**e**–**h**) growth of the axillary buds of the 2nd leaf at 1, 2, 3 and 4 weeks after topping; (**l**) growth of axillary buds of the 3rd leaf at 1, 2, 3 and 4 weeks after topping; (**m**) length of the axillary buds at the 1st, 2nd and 3rd leaves after topping and treated with lanolin/50 µM dexamethasone of positive transgenic plants.

To compare the differences between applied and unapplied sites on one plant, we compared and photographed the axillary buds of the 1st leaf that was applied with lanolin containing 50 µM DEX after topping during the vigorous growth period, i.e. Figure 5a–d, with the axillary buds of the 2nd and 3rd leaves at different time points after topping (Fig. 5a–m). The 50 µM DEX concentration inhibited the growth of axillary buds at the application site after topping of transgenic tobacco plants, while axillary buds of other leaves developed normally owing to the removal of apical dominance. These results shown that the bud development could be accurately inhibited by hormone application in transgenic tobacco plants.

### Preliminary toxicity studies of transgenic diphtheria protein containing plants

To demonstrate whether the diphtheria toxin could be transported to other non-intended plant parts and be toxic to other parts of the plant outside the site of application, fluorescence quantitative PCR was used to determine the expression level of DTA in different tissues including axillary buds of the 1st, the 2nd and 3rd leaves, as well as the 1st leave, the stems, and roots 4 h after treatment.

**Figure 6:**
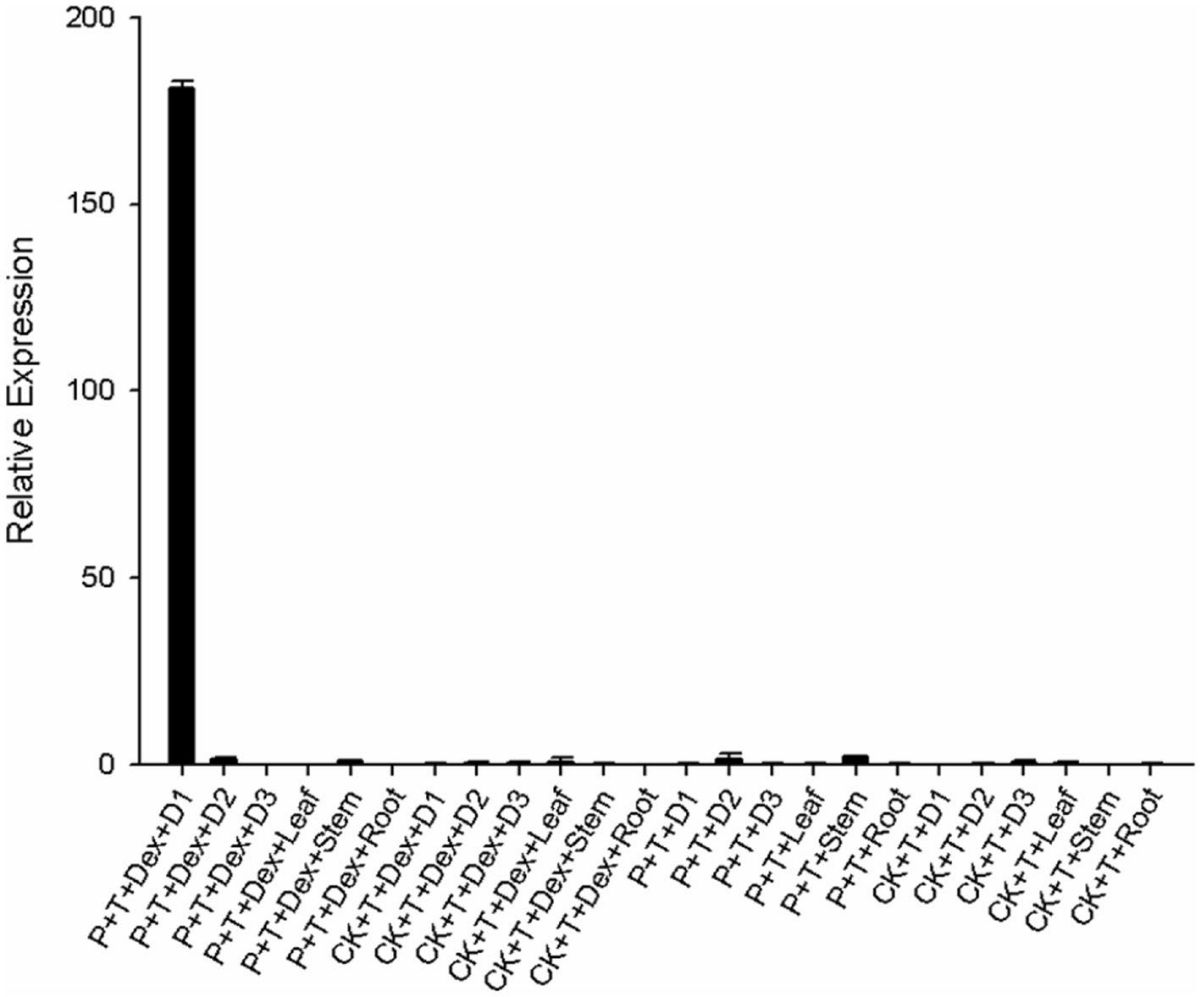
Transcriptional levels of *DTA* gene on the axillary buds of the 1st, 2nd and 3rd leaves, the 1st leave, the stems and roots after topping of positive transgenic plants/control plants applied with/without 50 µM DEX. P: positive transgenic plants; T: topping; Dex: 50 µM dexamethasone; D1: axillary buds of the 1st leaves; D2: axillary buds of the 2nd leaves; D3: axillary buds of the 3rd leaves.

**Figure 7:**
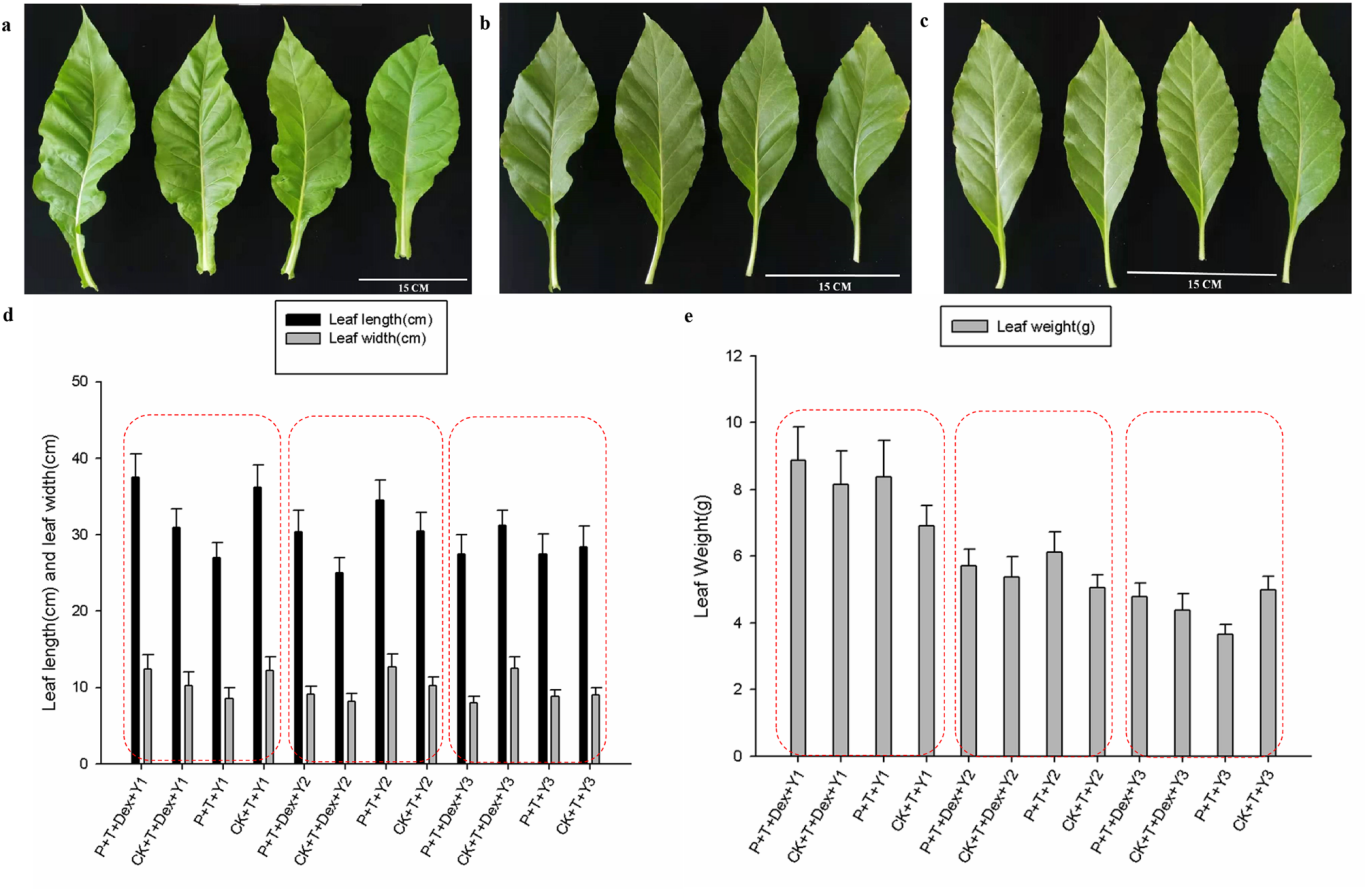
The leaf length, leaf width and leaf weight of the 1st, 2nd and 3rd leaves after topping of positive transgenic plants/control plants applied with/without 50 µM DEX. P: positive transgenic plants; T: topping; Dex: 50 µM dexamethasone; Y1: the 1st leaves of the application site; Y2: the 2nd leaves; Y3: the 3rd leaves. (**a**) Comparison of the 1st leaf after topping, from left to right: positive transgenic plants treated with 50 µM dexamethasone, positive transgenic plants treated with lanolin, control plants treated with 50 µM dexamethasone, control plants treated with lanolin; (**b**) comparison of the 2nd leaf after topping, from left to right: positive transgenic plants treated with 50 µM dexamethasone, positive transgenic plants treated with lanolin, control plants treated with 50 µM dexamethasone, control plants treated with lanolin; (**c**) comparison of the 3rd leaf after topping, from left to right: positive transgenic plants treated with 50 µM dexamethasone, positive transgenic plants treated with lanolin, control plants treated with 50 µM dexamethasone, control plants treated with lanolin; (**d**) comparison of the leaf length and leaf width of the 1st, 2nd and 3rd leaves after topping of positive transgenic plants/control plants applied with/without 50 µM DEX; (**e**): comparison of the leaf weight of the 1st, 2nd and 3rd leaves after topping of positive transgenic plants/control plants applied with/without 50 µM DEX.

The results show that the DTA expression level of the axillary bud at the 1st leaf from positive transgenic seedlings supplemented with 50 µM DEX treatment after 4 h was nearly 180-fold higher than that from the other tissues, while transcriptional level of DTA could not be detected in other parts of positive transgenic seedlings/control seedlings with/without 50 µM DEX treatment (Fig. 6). Meanwhile, the leaf length, leaf width and leaf weight of plants with different treatments after topping were measured separately. As shown in Fig. 7, the size and biomass of leaves exist no obvious difference between positive transgenic seedlings and control seedlings after different treatments.

These results suggest that the DTA acts only at the intended site and cannot be transferred to other unapplied sites in plants, and the DTA toxin that is precisely restricted to the axillary bud of the 1st leaf, does not disturb the size and yield of tobacco leaves.

## Discussion

Topping treatment is essential in the field production of tobacco in order to improve the quality of harvested leaves, however, application of suckercide is the most common method for inhibiting the sprout of axillary buds and consequent cause unnecessary nutrition consumption, which will lead to pesticide residues and environmental pollution. Like many other crops that ideal plant architecture can be obtained by regulating genes associated with branch development ^23–26^, the research on control of tobacco axillary buds is urgently needed.

At present, utilizing the expression of toxin genes driven by inducible promoters or tissue-specific promoters has been proved to be effective methods to control plant sterility^27^, seed germination ^28^ and insect-resistant^29^. However, there is no report on utilizing the toxin genes to control of plant branching. In this study, An effective toxin gene control system for tobacco based on the DEX induction was developed, the transgenic tobacco plants exhibit growth retardation in axillary buds. Besides, although studies have shown that dexamethasone and diphtheria toxin have some toxic effects, our experiments have confirmed that they will not be transported to other organs except the application site, nor do they affect the biomass of organs other than the application site^30–32^.

Previous analysis of various DTA mutants verified that the inability of CRM45, a nontoxic chain-termination mutant of DTA lacking the C-terminal region, to bind to cells gave indication that this missing region was involved in receptor binding^33–35^, which provides evidence that we can reduce the toxicity of DTA by removing 24 amino acids at the C-terminal end of the Diphtheria toxin-A protein. Our research provides a new insight to further control axillary buds in tobacco via inducible system as well as a reference for the branches control of tomato, cotton, and other crops.

The control of tobacco axillary buds after topping in field production may be carried out from the following aspects in the future, develop alternative, cheaper chemical inducers; explore axillary bud specific promoters and construct the recombinant vector of toxin gene driven by specific promoter; analyzing the patterns of axillary bud development in the early stage after topping and obtain the tobacco resources that can remain dormant after topping.

## Supplementary Information

Supplementary Information 1.
