# Supplementary figures and images for: Control of axillary bud growth in tobacco through toxin gene expression system
Source: Sci Rep. 2021 Sep 1;11:17513. doi: 10.1038/s41598-021-96976-3 (PMC8410782; doi:10.1038/s41598-021-96976-3)

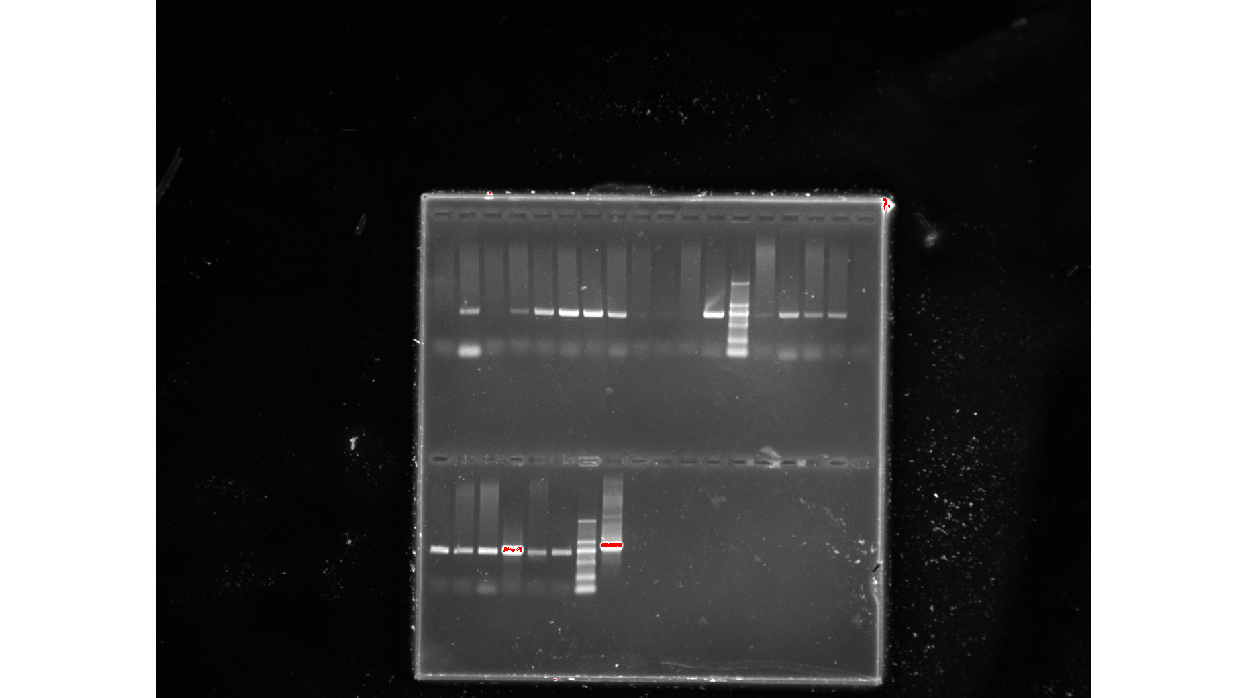

Supplement: Supplementary file 1 — Supplementary Information 1. [file 41598_2021_96976_MOESM1_ESM.tif]
